# Central and Peripheral Mechanisms Underlying Physiological and Drug-Induced Fluctuations in Brain Oxygen in Freely-Moving Rats

**DOI:** 10.3389/fnint.2018.00044

**Published:** 2018-10-02

**Authors:** Eugene A. Kiyatkin

**Affiliations:** In-Vivo Electrophysiology Unit, Behavioral Neuroscience Branch, National Institute on Drug Abuse—Intramural Research Program, National Institutes of Health, Department of Health and Human Services (DHHS), Baltimore, MD, United States

**Keywords:** brain metabolism, amperometry, glucose, neuronal activity, cerebral blood flow, heroin, fentanyl, oxycodone

## Abstract

The goal of this work is to consider physiological fluctuations in brain oxygen levels and its changes induced by opioid drugs. This review article presents, as a comprehensive story, the most important findings obtained in our laboratory by using high-speed amperometry with oxygen sensors in awake, freely moving rats; most of these findings were separately published elsewhere. First, we show that oxygen levels in the nucleus accumbens (NAc) phasically increase following exposure to natural arousing stimuli. Since accumbal neurons are excited by arousing stimuli and NAc oxygen levels increase following glutamate (GLU) microinjections in the NAc, local neural activation with subsequent cerebral vasodilation appears to mediate the rapid oxygen increases induced by arousing stimuli. While it is established that intra-cerebral entry of oxygen depends on brain metabolism, physiological increases in NAc oxygen occurred more rapidly than increases in metabolic activity as assessed by intra-brain heat production. Therefore, due to neural activation and the subsequent rise in local cerebral blood flow (CBF), the brain receives more oxygen in advance of its metabolic requirement, thus preventing potential metabolic deficits. In contrast to arousing stimuli, three opioid drugs tested (heroin, fentanyl and oxycodone) decrease oxygen levels. As confirmed by our recordings in the subcutaneous space, a densely vascularized location with no metabolic activity of its own, these decreases result from respiratory depression with subsequent fall in blood oxygen levels. While respiratory depression was evident for all tested drugs, heroin was ~6-fold more potent than oxycodone, and fentanyl was 10-20-fold more potent than heroin. Changes in brain oxygen induced by respiratory depression appear to be independent of local vascular and blood flow responses, which are triggered, via neuro-vascular coupling, by the neuronal effects of opioid drugs.

## Introduction

The brain is one of the heaviest consumers of oxygen in the body (Siesjo, 1978; Rolfe and Brown, 1997) and oxygen consumption increases during neuronal activation (Fox and Raichle, 1986; Fellows and Boutelle, 1993; Attwell et al., 2010). High metabolic activity of the central neurons requires continuous and efficient intra-brain delivery of oxygen from arterial blood that occurs via a concentration-dependent diffusion (Attwell et al., 2010). It is generally believed that oxygen entry into brain tissue is enhanced due to neuronal activation that dilates cerebral vessels and increases local cerebral blood flow (CBF; Fox and Raichle, 1986; Martin et al., 2006; Attwell et al., 2010; Paulson et al., 2010; Lecrux and Hamel, 2011; Muoio et al., 2014). Oxygen entry into brain tissue also depends on oxygenation of arterial blood, which is determined by respiratory activity, a highly variable physiological parameter that is affected by activity state, behavior and different drugs. All these dynamic changes in metabolic oxygen consumption and its entry from arterial blood affect extracellular oxygen levels in brain tissue, an important homeostatic parameter critical for maintaining proper neuronal activity and neural functions. Due to these dynamic and potentially opposing influences, it remains unclear how the balance between oxygen consumption and its entry from arterial blood is maintained under physiologically relevant conditions, how it is changed during neural activation elicited by natural arousing stimuli, and how it is affected by drugs which directly affect the CNS and respiration.

The goal of this work is to consider physiological fluctuations in brain oxygen levels and their changes induced by opioid drugs that directly affect the CNS and respiration. This work is based primarily on series of our recent experiments obtained by using oxygen microsensors coupled with high-speed amperometry in freely-moving rats. Although this technology cannot provide information on oxygen consumption resulting from brain metabolic activity, it is a valid tool to examine physiological and drug-induced fluctuations in brain oxygen in discrete brain areas and other bodily locations. To better understand the mechanisms governing oxygen entry into brain tissue under different experimental conditions, we also consider our data on fluctuations in brain glucose, another critical metabolic parameter. Like oxygen, glucose arrives to the brain from arterial blood based on its concentration gradient via GLUT-1 transporters (Duelli and Kuschinsky, 2001), and its entry into brain tissue shares many common features with that of oxygen. Finally, to examine the possible relationships between changes in oxygen and brain metabolism, we consider our thermorecording data, which provide critical information on physiological and drug-induced changes in brain temperature and brain metabolic activity as assessed by intra-cerebral heat production.

Voltammetric oxygen detection have a long history (Davies and Brink, 1942; Davies and Bronk, 1957; Clark et al., 1958), and oxygen sensors have been used previously with both amperometry (Lowry et al., 1997; Bolger and Lowry, 2005; Bolger et al., 2011; Francois et al., 2012; Kealy et al., 2013; Lyons et al., 2016) and cyclic voltammetry (Wang and Venton, 2016). Our work is an extension of these original studies and further application of this technology for neuroscience and neuropharmacology.

## Physiological Fluctuations in Brain Oxygen and Their Underlying Mechanisms

In mammals, oxygen is constantly delivered to the brain and other organs by arterial blood, and its gradient-dependent diffusion into brain tissue depends on oxygen content in the inspired air. The amount of inspired air depends on respiratory activity, which determines the oxygen transfer from the external medium to the lung’s vessels. Therefore, extracellular levels of oxygen in brain tissue depend on oxygen content in the external medium and this basic dependence has been demonstrated in numerous studies, where brain oxygen levels either increased or decreased when the recorded animal was exposed to air with high or low oxygen content, respectively (Bazzu et al., 2009; Bolger et al., 2011; Li et al., 2011; Kealy et al., 2013). Brain oxygen levels also depend on the efficiency of respiratory activity and subsequent changes in blood oxygen levels, and this physiological parameter can be changed within wide limits during physiological and behavioral activation and following exposure to certain drugs that affect respiration. While the respiration-dependent changes in blood oxygen content will definitely modulate oxygen entry into brain tissue, it is quite difficult to distinguish the contribution of this factor due to simultaneous changes in neuronal activity, brain metabolism, and the tone of cerebral vessels, which all are affected by physiological and drug stimuli. While these slowly-acting influences related to oxygen content in the external medium and the efficiency of respiration affect oxygen levels in brain tissue, more complex phasic changes in brain oxygen levels occur after exposure to sensory and arousing stimuli, which elicit changes in neuronal activity, brain metabolism, and cardio-vascular functions, including perfusion pressure and the tone of peripheral and cerebral vessels.

To examine physiological fluctuations in brain oxygen levels, we used Pt-Ir oxygen sensors (Pinnacle Technology) coupled with high-speed amperometry in awake, freely moving rats (Solis et al., 2017a). This technology was used to examine how natural arousing stimuli affect oxygen levels in the nucleus accumbens (NAc)—a critical structure for sensorimotor integration and behavioral regulation (Mogenson et al., 1980; Wise and Bozarth, 1987; Di Chiara, 2002).

**Figure 1:**
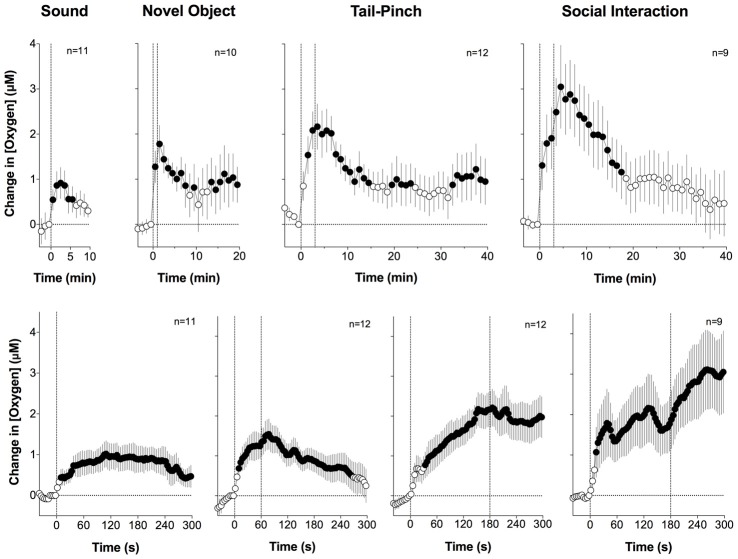
Changes in nucleus accumbens (NAc) oxygen levels induced by arousing stimuli in freely-moving rats. Top graphs show mean (± SEM) changes relative to pre-stimulus baseline (= 0 μM) analyzed with a slow, 1-min time resolution and bottom graphs show changes assessed at a high, 4-s time resolution. Values significantly different from pre-stimulus baseline (*p* < 0.05, Fisher F-test) are shown as filled symbols. n indicates the number of averaged tests. The first dotted vertical line shows the stimulus onset, the second vertical line shows the stimulus offset. Original data were presented in Solis et al. (2017a).

When analyzed with slow (1-min) time resolution, we found that all arousing stimuli significantly increased NAc oxygen levels (Figure 1). The increase was weakest in amplitude and duration after auditory stimulation, moderate after novel object presentation, and greatest and most prolonged during tail-pinch and social interaction with a male conspecific. When calculated as a percent change relative to the resting oxygen baseline, these increases were relatively small in magnitude (5%–15%). Since maximal changes for all stimuli occurred during the first minute after the stimulus onset, we used high-resolution analyses (4-s bins) to represent oxygen dynamics. In this case, we found that oxygen responses are very rapid, with latencies to increase corresponding to 8–12 s after the stimulus onset, and distinct for each stimulus. The increase was weakest and monotonic after auditory stimulation, whereas the presentation of a novel object resulted in a two-phasic oxygen increase, with the second rise occurring immediately after the novel object was removed from the cage. Tail-pinch induced a continuous increase in oxygen levels that began from the onset of stimulation, continued while the rat actively bit or chewed on the clothespin used for the tail-pinch, and slowly returned to baseline when the clothespin was removed from the rat’s tail. The fastest and largest oxygen increase occurred at the start of and during social interaction. In this case, oxygen levels also displayed a second, rapid but much smaller, increase following guest rat removal, which corresponded to increased cage exploration by the recorded rat.

Rapidity of brain oxygen responses found in our experiments suggests the involvement of a neural mechanism, a stimulus-induced neuronal activation, as their cause. It is well known that dorsal and ventral striatal neurons have low impulse activity in awake rats during quiet resting conditions but are phasically excited following exposure to various arousing stimuli (Carelli and West, 1991; Kiyatkin and Rebec, 1996, 1999; Rebec, 2006). These neuronal excitations, via a neurovascular coupling mechanism, could lead to local vasodilation followed by the rapid rise in local CBF that phasically enhances oxygen entry into brain tissue. To directly test this mechanism, we examined changes in NAc oxygen induced by local micro-injections of glutamate (GLU) near the oxygen detecting site. It is well established that GLU delivery either by local microinjection or iontophoresis induces dose-dependent increases in impulse activity of accumbal neurons (Kiyatkin and Rebec, 1999). If neuronal activation is responsible for phasic NAc oxygen increases, oxygen levels should increase following local GLU microinjections.

**Figure 2:**
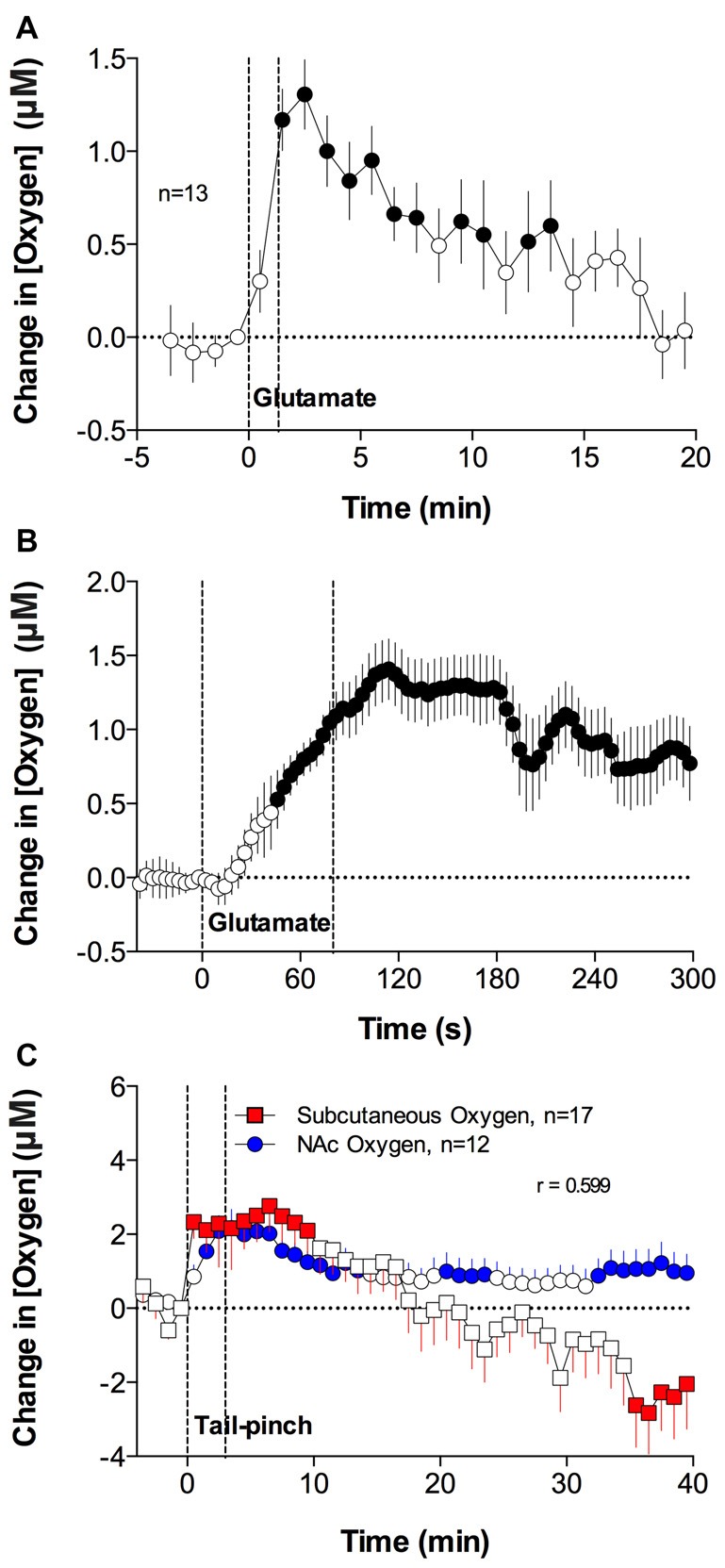
Mean (± SEM) changes in NAc oxygen levels induced by local microinjections of glutamate (GLU) near the oxygen-detecting site **(A,B)**. Comparison of changes in oxygen levels in the subcutaneous space and NAc induced by 3-min tail-pinch **(C)**. Durations of GLU microinjection **(A,B)** and tail-pinch **(C)** are shown as vertical dotted lines. *n* indicates the number of averaged tests. The technical details of combined neurochemical recording and drug microinjections have been presented elsewhere (Kiyatkin and Lenoir, 2012; Solis et al., 2017a). In brief, L-GLU (as a monosodium salt) was delivered in 1–5 mM solutions (0.2–0.5 μl) via small, stainless steel tubing (120 μm internal diameter), with its tip located within ~200–400 μm from the oxygen recording site. In experiments assessing oxygen changes in subcutaneous space, cannulae were implanted subcutaneously in the medio-frontal area of the rat’s head. Values significantly different from baseline are shown as filled symbols. Original data were published in Solis et al. (2017a).

As shown in Figure 2, this was the case. When injected at the optimal concentration, NAc oxygen levels increase following GLU microinjection. In contrast to oxygen responses elicited by arousing stimuli, GLU-induced oxygen responses were more transient, exhibited variable latencies, and were of relatively low magnitudes (0.5–2 μM) that were similar or lower than those seen with natural arousing stimuli. When analyzed as a mean of all GLU injections at the same dose, the oxygen increase was highly significant, occurred with ~40-s latency after the injection onset, peaked 30–40 s after injection offset, and slowly decreased towards baseline thereafter.

Arousing and stressful stimuli, including those used in our studies, induce sympathetic activation (Cannon, 1929; Silverthorn, 2004), thus increasing respiration rate, the volume of air inspired, and brain oxygen uptake (Bedford et al., 1979; Kabir et al., 2010) and resulting in increased saturation of hemoglobin and greater oxygen diffusion into brain tissue from dilated cerebral vessels. Although local neural activation definitely contributes to brain oxygen increases elicited by arousing stimuli, increases in blood oxygen levels due to respiratory activation could be another contributor. An optimal approach to test how changes in blood oxygen content affect fluctuations in NAc oxygen is to monitor oxygen levels directly in arterial blood. This approach, however, is technically not feasible in freely moving rats. Therefore, we sought to overcome this obstacle by assessing changes in oxygen levels in the subcutaneous space—a densely vascularized area with limited or very low levels of metabolic activity of its own. Through using direct, simultaneous measurements, it has been shown that glucose levels in subcutaneous space tightly correlate with glucose levels in arterial blood, suggesting that this recording location could serve as a useful proxy for arterial blood (Moon et al., 2013). Our expectation was that the changes in oxygen current recorded from this location would reflect, with a possible latency, changes in oxygen occurring in arterial blood.

We found that arousing stimuli, including tail-pinch and social interaction, significantly increase oxygen levels in the subcutaneous space (Figure 2C); their changes generally paralleled changes in NAc oxygen and they displayed a significant correlation. While comparable in magnitude and duration, these oxygen changes were more variable than those seen in the NAc oxygen. Therefore, in addition to a neuro-vascular coupling mechanism, increases in blood oxygen levels occurring due to sympathetic activation provide a certain, albeit minor, contribution to increases in brain oxygen levels elicited by arousing stimuli.

## Relationships Between Physiological Fluctuations in NAc Oxygen and Glucose

Similar to oxygen, glucose enters brain tissue from arterial blood by gradient-dependent diffusion via GLUT-1 transporters (Duelli and Kuschinsky, 2001). In contrast to oxygen, for which blood levels are variable and dependent on respiration, glucose is normally present in the blood at relatively stable levels, but is able to dose-dependently increase by iv glucose administration (Lowry et al., 1998; Kiyatkin and Lenoir, 2012) and during glucose-drinking behavior (Wakabayashi and Kiyatkin, 2015), and decreased dramatically by insulin administration (Kealy et al., 2015). In contrast to oxygen that is contained in extracellular space at low, 10–20 μM levels and which could increase by arousing stimuli by only 10%–20% above a quiet-resting baseline, the basal levels of glucose are much higher (0.5–1.0 mM; Fellows et al., 1992; Lowry et al., 1998; McNay et al., 2001; Kiyatkin and Lenoir, 2012; Kealy et al., 2015), and they could easily be doubled following iv glucose administration, without any obvious behavioral effects.

**Figure 3:**
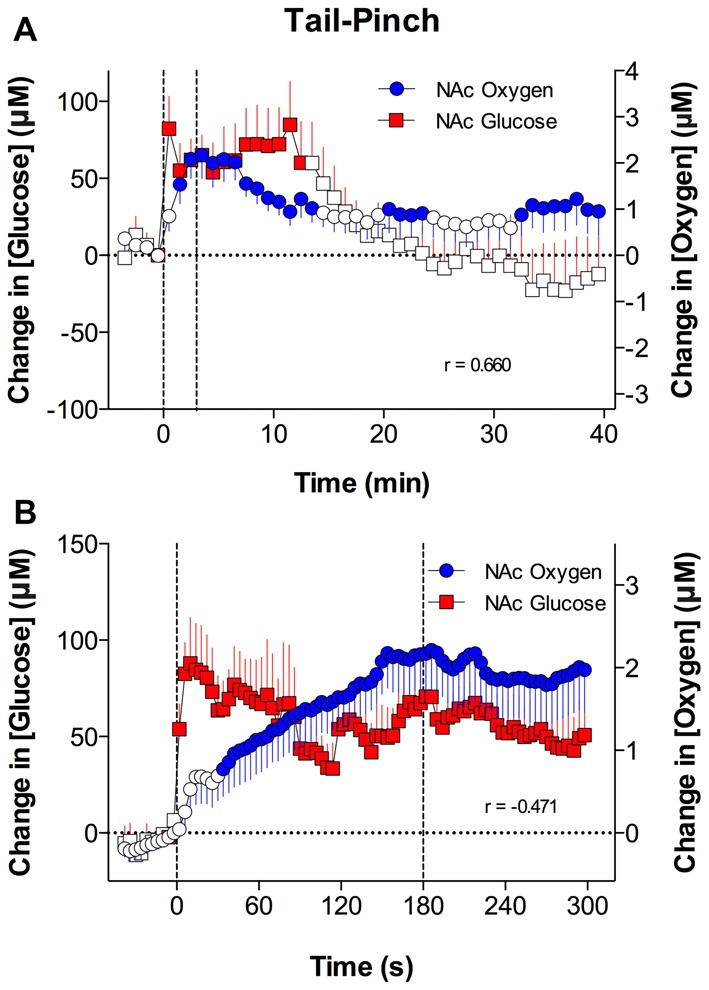
Relationships between changes in NAc oxygen and glucose induced by tail-pinch. Top graphs **(A)**, show mean (± SEM) changes in concentrations of oxygen and glucose analyzed with slow time resolution (1-min bins) and bottom graph **(B)** shows changes analyzed with rapid time resolution (4-s bins). Vertical dotted lines show onset and offset of tail-pinch. Each graph shows coefficients of correlation (r). Original data were published in Solis et al. (2017a).

Similar to oxygen, glucose entry into brain tissue is phasically increased due to proximal neuronal activation, which induces local vasodilation and enhances CBF (Fellows et al., 1992; Silver and Erecinska, 1994; Attwell et al., 2010; Mergenthaler et al., 2013). Due to commonality of basic mechanisms governing intra-brain entry of oxygen and glucose, we hypothesized that the patterns of their changes in the NAc would share important similarities. As shown in Figure 3, this was the case. Similar to oxygen, NAc glucose levels increased following arousing stimuli (Kiyatkin and Lenoir, 2012; Wakabayashi and Kiyatkin, 2015). While the absolute magnitude of NAc glucose increases were proportionally larger than those for oxygen, the response magnitude as a percent change relative to the baseline (500–800 μM vs. 10–20 μM for glucose and oxygen, respectively) was comparable for both parameters (10%–20%). When data were analyzed with a slow, 1-min time resolution, the changes in oxygen and glucose had a similar pattern and significantly correlated during both tail-pinch and social interaction (Figure 3). However, glucose levels displayed maximal increases and peaked during the first minute of stimulation, whereas oxygen levels increased more tonically and peaked after the end of the tail-pinch and social interaction stimuli.

However, when the data were analyzed with high temporal resolution, changes in NAc oxygen and glucose had drastic differences in the response time-course (Figure 3). In contrast to the rapid, accelerating rise in oxygen, glucose levels peaked phasically at ~10 s after the start of the tail-pinch and social interaction stimuli and then slowly returned to the pre-stimulus baseline. Thus, the increases in NAc glucose are clearly more phasic and more transient than the oxygen increases. Due to these differences in the rapid time-course, changes in oxygen and glucose negatively correlated; when glucose started to decrease after the initial peak, oxygen continued to rise.

## Relationships Between Changes in NAc Oxygen, Brain Temperature, and Brain Metabolic Activation

Neural activity is highly energy-consuming and all of the energy used from brain metabolism is finally transformed into heat (Hodgkin, 1967; Ritchie, 1973; Siesjo, 1978; Schmidt-Nielsen, 1997; Sokoloff, 1999). As shown previously (see Kiyatkin, 2010 for review), any arousing stimulus presented to awake rats elicits moderate increases in brain temperature that depend on two primary mechanisms: (1) an increase in intra-cerebral heat production due to metabolic brain activation; and (2) a decrease in heat loss to the external environment due to peripheral vasoconstriction. Since metabolic brain activation increases intra-cerebral heat production, we first examined the relationship between changes in NAc oxygen and temperature.

**Figure 4:**
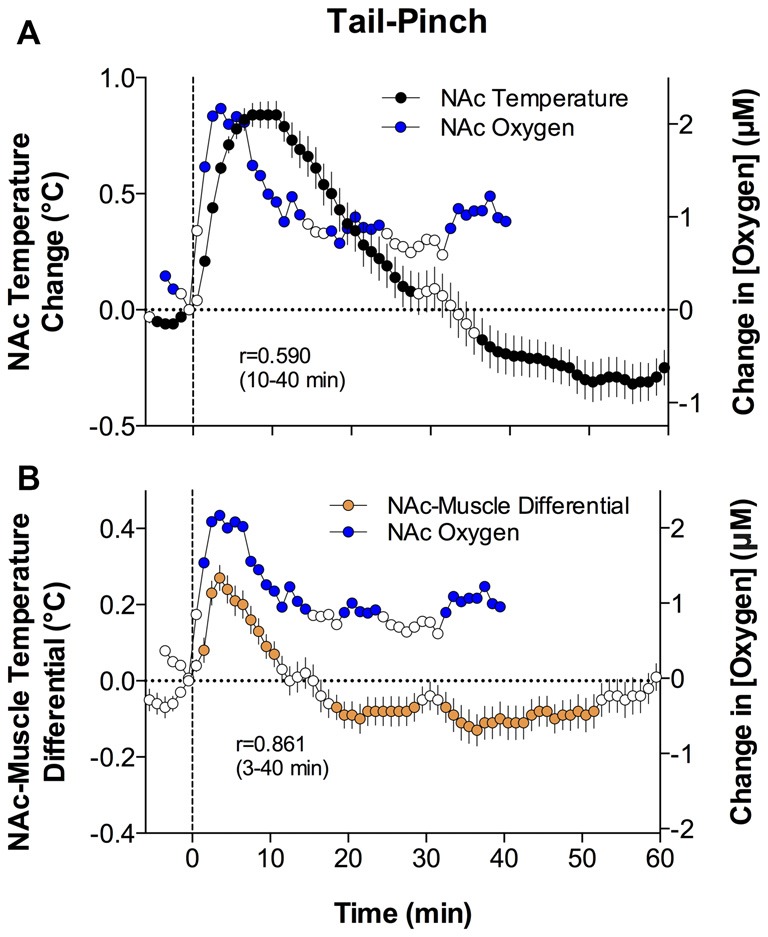
Relationships between changes in NAc oxygen and temperature parameters induced by tail-pinch. Panel **(A)** shows mean (± SEM) changes in NAc oxygen concentration (blue circles) and NAc temperature (black circles) analyzed with slow, 1-min time resolution. Panel **(B)** shows mean (± SEM) changes in NAc oxygen concentration (blue circles) and the NAc-Muscle temperature differential (orange circles) analyzed with slow, 1-min time resolution. Values significantly different from baseline are shown as filled symbols. Vertical hatched lines show onset of tail-pinch. Each graph shows coefficients of correlation (r) for compared parameters during specified time intervals following the onset of tail-pinch. Original data were published in Solis et al. (2017a).

As shown in Figure 4A, both oxygen levels and temperature recorded from the NAc increased during tail-pinch; these parameters significantly correlated (*r* = 0.590, *p* < 0.01). However, oxygen increases occurred more rapidly, more strongly, and peaked at an earlier time than changes in brain temperature. Oxygen levels surged during the first minute of stimulation, when brain temperature had only begun to increase and was still close to baseline. Then, oxygen levels continued to increase during the next 2–3 min and showed a strong direct correlation with brain temperature. This correlation disappeared from ~8 min after stimulus onset, when both parameters began to decrease toward baseline.

Although brain temperature is an important homeostatic parameter and its changes are related to metabolic activity, changes in brain temperature also depend on the temperature of arterial blood. Since the temporal muscle is a non-locomotor muscle and it receives its arterial blood supply from the carotid artery like the brain does, the difference between brain and muscle temperature (or the brain-muscle differential) eliminates the influence of arterial blood temperature on brain temperature, thus allowing us to reveal the component of the temperature response that is related to intra-brain heat production resulting from metabolic brain activation (Kiyatkin, 2010).

Similar to oxygen, brain-muscle differentials increased during tai-pinch, showing a tight direct correlation (*r* = 0.861, *p* < 0.0001) for the entire analysis interval apart from the short time intervals following the stimulus onset (Figure 4B). During this initial time interval following the onset of tail-pinch, oxygen levels rapidly and strongly increased, preceding weaker and more delayed increases in NAc-muscle temperature differentials. Therefore, oxygen enters the brain tissue preceding metabolic activation as assessed by intra-brain heat production.

Since all arousing stimuli, including those used in this study, variably increase brain temperature and increases in temperature facilitate the diffusion of gases in liquids (first Fick’s law; Longsworth, 1954; Han and Bartels, 1996), stimuli-induced elevations in brain temperature should enhance oxygen diffusion into brain tissue, thus also contributing to NAc oxygen increases. This contribution, however, appears to be minimal compared to contributions related to changes in cerebral vascular tone due to neuronal activation.

## Changes in Brain Oxygen Induced by Opioid Drugs

Opioids are widely used as therapeutic drugs to alleviate pain of different origins. In addition to their pain-relieving effects, opioid drugs have strong addictive potential, predisposing individuals for their repeated, non-medical use. In addition, opioid drugs have a number of side effects, including hypoactivity, sedation, inhibition of gastro-intestinal activity, and respiratory depression (Jaffe et al., 1997; Simon, 1997; Baud, 2009). The latter effect is especially dangerous, being responsible for the development of acute brain hypoxia, coma and lethality following overdose of these drugs. Due to the critical role of respiratory depression in mediating acute life-threatening effects of opioid drugs, we examined changes in brain oxygen induced by three representative opioids: oxycodone, heroin and fentanyl (Solis et al., 2017b,c, 2018a,b).

Oxycodone is one of the most prescribed opioid drugs. In addition to its therapeutic use as an analgesic, non-medical use of oxycodone has dramatically increased during recent years. Heroin is currently not used for therapeutic purposes, but it appears to be the most well-known drug of abuse, which has rapid and exceptionally strong psychoactive and physiological effects. Fentanyl is used extensively in humans for general anesthesia and analgesia (Peng and Sandler, 1999), but in recent years it has emerged as a drug of abuse, often used in combination with heroin (Compton et al., 2016; McLaughlin, 2017). Since fentanyl is 20–40× more potent than heroin (Wade et al., 2015), its illicit use can result in adverse health effects, including death during overdose (Compton et al., 2016; Suzuki and El-Haddad, 2017).

**Figure 5:**
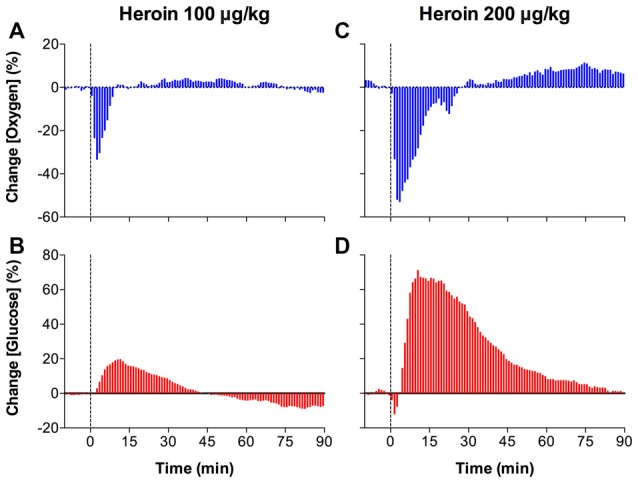
Mean (± SEM) changes in NAc levels of oxygen **(A,C)** and glucose **(B,D)** induced by intravenous heroin injections at two low doses. Data are analyzed with slow, 1-min time resolution and are expressed in percent vs. pre-injection baseline (= 0). Vertical dotted lines show the onset of injection. Original data were published in Solis et al. (2017c).

In contrast to oxygen increases elicited by natural arousing stimuli, iv heroin induced a rapid and strong decrease in NAc oxygen levels, suggesting acute brain hypoxia (Figure 5). At a low dose (0.1 mg/kg) optimal for self-administration in rats, the hypoxic effect was rapid, appearing within the first minute of drug administration, strong with a ~30% drop from baseline, but relatively transient in its duration (Figure 5A). At a higher heroin dose (0.2 mg/kg) within the range injected by experienced drug users, this effect greatly increased in magnitude (~50% drop from baseline) and duration (~20 min), suggesting robust brain hypoxia (Figure 5C). At this dose, the effect of heroin was biphasic, with a slight increase in NAc oxygen levels after a strong decrease seen immediately after iv injection. In contrast to oxygen, heroin dose-dependently increased NAc glucose levels (Figures 5B,D). These effects were more prolonged than those of oxygen and they were stronger in absolute amplitude (increases to 180 and 350 μM for 0.1 and 0.2 mg/kg, respectively), but comparable in terms of percent change. Importantly, the heroin-induced oxygen decrease was rapid (onset latency ~40 s) and peaked at 120–180 s, but the glucose rise developed with a definite latency (~3–4 min), suggesting that this change is dependent on and secondary to brain hypoxia that results from respiratory depression. In addition to decreasing oxygen levels in the brain tissue, respiratory depression results in diminished outflow of CO_2_ to the external environment, thus resulting in its accumulation in arterial blood and increase in CO_2_ levels in brain tissue. Since CO_2_ is known to cause cerebral vasodilation (Schmidt and Kety, 1947; Kontos, 1981; Battisti-Charbonney et al., 2011), the heroin-induced rise in brain CO_2_ levels coupled with metabolic acidosis and accumulation of other metabolites appear to be responsible for dilation of cerebral vessels, which results in increased CBF and enhanced glucose entry into the brain tissue.

**Figure 6:**
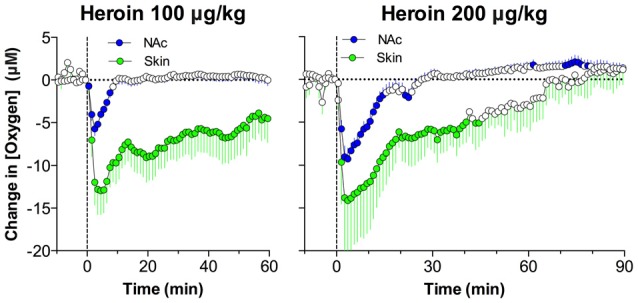
Comparison of changes in oxygen levels in the NAc and subcutaneous space induced by intravenous heroin injections at two low doses. Data are shown as changes in mean (± SEM) concentrations analyzed at slow, 1-min time resolution. Vertical hatched lines show the onset of injection. Since absolute values of dissolved oxygen in the subcutaneous space are larger than those in the NAc, heroin induced larger oxygen concentration decreases (in μM) in this peripheral location. When calculated as relative change, the amplitude of oxygen decreases was similar in both recording locations. Original data were published in Solis et al. (2017c).

While we observed that heroin induces shallow breathing, suggesting respiratory depression, this mechanism was confirmed by oxygen measurements in the subcutaneous space. As shown in Figure 6, heroin significantly decreased oxygen levels in the subcutaneous space; this effect was dose-dependent and larger than that in the NAc. In contrast to the NAc, where the oxygen decrease was transient, in the subcutaneous space it was much longer in duration. Therefore, respiratory depression and a decrease in blood oxygen levels is the primary cause of the oxygen drop in brain tissue.

**Figure 7:**
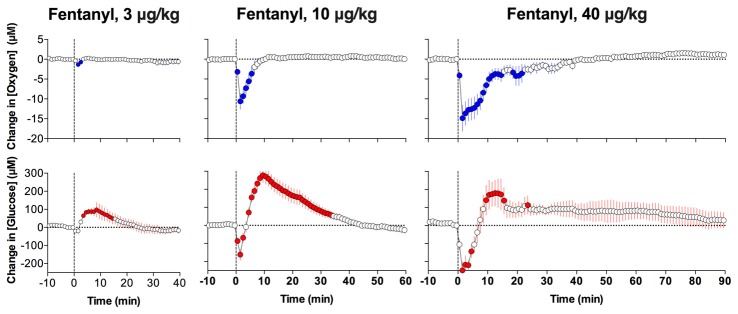
Mean (± SEM) changes in NAc oxygen (top graphs) and glucose (bottom graphs) concentrations induced by intravenous fentanyl injections at different doses. Data were analyzed with slow, 1-min time resolution. Vertical hatched lines show onset of injection. Original data were published in Solis et al. (2018b).

Fentanyl also decreased NAc oxygen levels (Figure 7); this effect appeared at very low doses and became stronger with dose increases. Similar to heroin, the effect was very rapid, appearing within the first minute and peaking at the second minute after the onset of iv injection. Fentanyl also increased glucose levels, but this effect was monophasic at low doses and became biphasic (transient decrease followed by an increase) at larger doses. Based on the strength of brain oxygen decreases, fentanyl is 10-20-fold stronger than heroin. It is currently unclear why glucose levels transiently drop after the injection, but a correlation seen at this time between oxygen and glucose levels suggest that the glucose drop could be a consequence of a deficit in oxygen, which is necessary for glucose oxidation and subsequent detection at the sensor.

Street heroin currently available to drug users is often contaminated by fentanyl. Due to fentanyl’s increased potency in terms of inducing respiratory depression (Yeadon and Kitchen, 1990; Dahan et al., 2005; Pattinson, 2008), intake of heroin-fentanyl mixtures may result in serious health complications, including comatose state and lethality (Compton et al., 2016; Suzuki and El-Haddad, 2017).

To mirror such a real-world scenario, in which an individual believing that he or she is consuming a standard dose of heroin is actually consuming a heroin sample laced with a smaller amount of fentanyl, we examined changes in NAc oxygen induced by heroin (0.4 mg/kg) contaminated with 10% fentanyl (0.04 mg/kg). These doses of both drugs are much lower than the LD50 assessed in rats (heroin: 15–20 mg/kg; Gable, 2004; Strandberg et al., 2006; fentanyl: 1–3 mg/kg; von Gunten et al., 2010) and are within the range of consumption by experienced drug users (see erowid.org).

**Figure 8:**
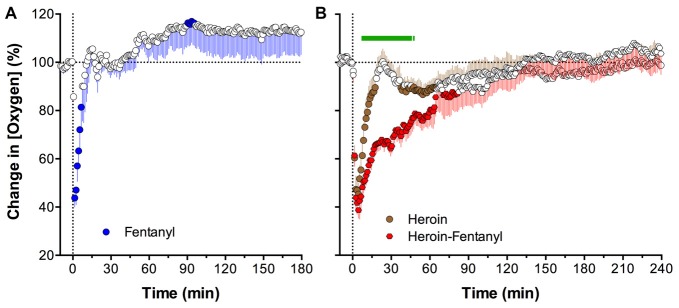
Mean (± SEM) changes in NAc oxygen levels induced by intravenous injections of fentanyl (**A**; 0.04 mg/kg), heroin (**B**; 0.4 mg/kg) and heroin laced with 10% fentanyl (**B**; 0.36 + 0.04 mg/kg, respectively). Values significantly different from baseline are shown as filled symbols. Green line in **(B)** shows time interval for which differences in changes between groups were significant. Vertical dotted lines show onsets of injections. Data were analyzed with slow, 1-min time resolution and they are expressed as a relative change vs. pre-injection baseline. Original data were published in Solis et al. (2017b).

As shown in Figure 8, both heroin and fentanyl injected individually induced strong but transient decreases in NAc oxygen levels which were rapid and relatively similar in magnitude (Solis et al., 2017b). A rapid and strong drop in NAc oxygen also occurred after administration of the heroin-fentanyl combination, but the response was much more prolonged than with each drug alone. As determined by the area under the curve for oxygen decrease, the effect of the heroin-fentanyl mixture was about 10-fold larger than fentanyl alone and ~5-fold larger than heroin alone. This prolongation of brain hypoxia is functionally important because brain cells may tolerate transient decreases in oxygen inflow, but are damaged to a greater extent when hypoxia is more prolonged (Hossmann, 1999).

**Figure 9:**
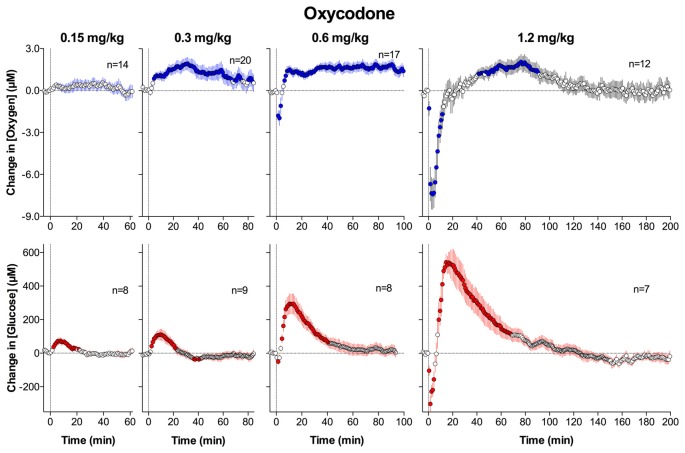
Mean (± SEM) changes in NAc levels of oxygen (top graphs) and glucose (bottom graphs) induced by intravenous injections of oxycodone at four different doses. Data were analyzed with slow, 1-min time resolution and they are expressed as absolute concentration change. Values significantly different from baseline are shown as filled symbols. Vertical hatched lines show onset of injection. *n* indicates the number of averaged tests. Original data were published in Solis et al. (2018a).

Recently, we extended this line of work to oxycodone, a potent analgesic drug, which is usually viewed as less dangerous in its acute effects than heroin or fentanyl (Solis et al., 2018a). In this study, we examined the effects of iv oxycodone at a wide range of doses on brain oxygen and glucose levels. In contrast to heroin, oxycodone at low and moderate doses (0.3–0.6 mg/kg) increased NAc oxygen levels (Figure 9). While significant, this effect was relatively weak, within 15% above the pre-injection baseline, but more tonic. At the highest dose (1.2 mg/kg), the effect became biphasic, with a transient decrease followed by an increase. A weak, transient drop in oxygen was also seen at the moderate oxycodone dose (0.6 mg/kg). When compared based on its ability to induce an oxygen drop following iv injection, oxycodone was about six times weaker than heroin and about 60–120-fold weaker than fentanyl. In other words, fentanyl and heroin are 6- and 60–120-fold more potent in inducing brain hypoxia than oxycodone.

Our parallel recordings with glucose sensors revealed that oxycodone dose-dependently increases NAc glucose levels (Figure 9), and that these increases are larger than those for oxygen (10%–70% above pre-injection baseline for 0.15–1.2 mg/kg, respectively). Only increases were seen at low doses, but the effect became biphasic with dose increases, with a transient dose-dependent fall immediately after injection. It is unclear why oxycodone at low and moderate doses increased levels of both oxygen and glucose, but this change could be related to cerebral vasodilation and increased CBF due to strong peripheral vasoconstriction, which was found through our parallel thermorecording experiments (Solis et al., 2018a).

## Conclusions and Functional Implications

Under physiological conditions, oxygen levels in neural tissue are maintained at relatively stable and low levels, providing adequate resources for ongoing metabolic activity of brain cells. These levels reflect a balance between two opposing forces: oxygen inflow from the arterial blood and oxygen consumption due to metabolic activity. Data shown in this review demonstrate that physiological activation elicited by natural arousing stimuli results in increases in NAc oxygen levels; these increases were very rapid, but relatively small in magnitude. While oxygen recordings in our studies were conducted in the NAc, it remains unclear to what extent changes occurring in this brain structure could be generalized to other brain structures. Converging evidence from the literature, our own glucose and temperature measurements suggest that these phasic increases in oxygen result from neuronal activation that triggers local vasodilation, which enhances oxygen diffusion from arterial blood into brain tissue. Based on our data, it is impossible to quantify oxygen use for metabolic activity, which should oppose oxygen entry from arterial blood. However, our data clearly indicate that oxygen levels during functional neural activation do not decrease but phasically increase. Importantly, increases in brain oxygen occur more rapidly than increases in metabolic activity as measured by intra-brain heat production. Therefore, due to neural activation elicited by arousing stimuli and the subsequent rapid rise in local CBF, the brain receives more oxygen in advance of its metabolic demand, thus preventing potential metabolic deficits.

In contrast to physiological stimuli that transiently increase oxygen levels in the NAc, opioid drugs decrease brain oxygen levels, suggesting hypoxia. These decreases differed for each of the three drugs tested in our studies (heroin, fentanyl, and oxycodone) and they increased in magnitude and duration with increases in drug doses. This hypoxic effect results from respiratory depression with subsequent decreases in blood oxygen levels. This effect is typical to all opioid drugs at high doses, and it appears to be independent of local vascular and blood flow responses occurring due to the neuronal effects of these drugs. Robust decreases in oxygen induced by large-dose opioid drugs in the NAc appear to be generalized to other brain structures as tested by our control recordings in the basolateral amygdala, a distantly located brain structures involved in regulating different brain functions (Solis et al., 2017b). While rats are less sensitive to oxygen deficits than humans and can tolerate even large but transient decreases in brain oxygen levels, acute hypoxia induced by large-dose opiate use could be fatal for humans.

## Author Contributions

The author confirms being the sole contributor of this work and approved it for publication.

## Conflict of Interest Statement

The author declares that the research was conducted in the absence of any commercial or financial relationships that could be construed as a potential conflict of interest.
